# Neural rhythmic underpinnings of intergroup bias: implications for peace-building attitudes and dialogue

**DOI:** 10.1093/scan/nsab106

**Published:** 2021-09-14

**Authors:** Jonathan Levy, Abraham Goldstein, Moran Influs, Shafiq Masalha, Ruth Feldman

**Affiliations:** Baruch Ivcher School of Psychology, Interdisciplinary Center, Herzliya 46150, Israel; Department of Neuroscience and Biomedical Engineering, Aalto University, Espoo 02150, Finland; Gonda Multidisciplinary Brain Research Center and Department of Psychology, Bar-Ilan University, Ramat Gan 5290002, Israel; Baruch Ivcher School of Psychology, Interdisciplinary Center, Herzliya 46150, Israel; Ono Academic College, Ono 55000, Israel; Baruch Ivcher School of Psychology, Interdisciplinary Center, Herzliya 46150, Israel; Child Study Center, Yale University, New Haven, CT 06520, USA

**Keywords:** intergroup conflict, intergroup bias, alpha rhythm, intergroup dialogue, conflict resolution, magnetoencephalography

## Abstract

Intergroup bias is a ubiquitous socio-cognitive phenomenon that, while sustaining human dependence on group living, often leads to prejudice, inequity, and violence; yet, its neural underpinnings remain unclear. Framed within the Israeli–Palestinian conflict and targeting youth, this study utilized magnetoencephalography to describe intrinsic neural oscillatory processes that represent the intergroup bias and may link with engagement in peacemaking in order to shed further light on the neural mechanisms underpinning intergroup conflict. Across the oscillatory spectrum, from very low to very high frequency bands, the only rhythm found to underlie the intergroup bias was the alpha rhythm. Alpha rhythm was continuously activated across the task and integrated a rapid perceptual component in the occipital cortex with a top-down cognitive-control component in the medial cingulate cortex. These components were distinctly associated with the real-life intergroup dialogue style and expressed attitudes that promote active engagement in peacemaking. Our findings suggest that the cortical alpha rhythm plays a crucial role in sustaining intergroup bias and addresses its impact on concrete intergroup experiences. The results highlight the need to provide opportunities for active peace-building dialogue to youth reared amidst intractable conflicts.

## Introduction

Individuals in human societies rely on group living for survival and thriving. This dependence has led to a biased assessment of their social environment; members of one’s social group (ingroup) are consistently perceived in more favourable terms as compared to members of other groups (outgroup) (Hewstone *et al.*, 2002). Although current social and political movements have gained some success in altering explicit negative attitudes towards outgroups, group-based prejudices, which often lead to violence and inequity, are still ubiquitous. For instance, while whites in the USA tend to express pro-black attitudes, they often support policies that are detrimental towards blacks (Charlesworth and Banaji, 2019). Similarly, whereas most Israelis express a desire for peace, they harbor implicit perceptions of Palestinians as hostile and threatening, which hinder their support for active peacemaking and paralyze the region in inertia and inactivity (Bar-Tal, 2001). Extant research has indicated that this gap marks a universal ‘implicit intergroup bias’ humans hold towards other groups (Ashburn-Nardo *et al.*, 2001; Kurdi *et al.*, 2019). This implicit bias can be used to indicate a subtle type of prejudice that is not consciously perceived (Sekaquaptewa *et al.*, 2003) and bypasses conscious awareness (Greenwald *et al.*, 2003). In certain contexts, such a bias can escalate into a violent or an even lethal battle (Plant and Peruche, 2005) and often perpetuates intergroup conflicts (Bar-Tal and Halperin, 2011a).

The Implicit Association Test (IAT) is the most widely used measure to evaluate implicit intergroup bias, with thousands of studies in the past decades (Greenwald and Lai, 2020). This test typically yields a slower response time in incongruent (IC) (e.g. outgroup good) *vs* congruent (C) (e.g. outgroup bad) trials. Such a behavioural effect, known as the ‘IAT effect’, typically reflects intergroup bias as has been shown in dozens of countries across the world (Kurdi *et al.*, 2019), including in the context of the Israeli–Palestinian conflict (Hassin *et al.*, 2009; Bruneau and Saxe, 2010; Danziger and Ward, 2010). Despite the prevalent use of the IAT, the real-life implications of intergroup bias remain highly equivocal (Blanton *et al.*, 2009; Oswald *et al.*, 2013, 2015; Greenwald *et al.*, 2019), and there is insufficient understanding of its neural underpinnings and the mental processes that contribute to its expression (Bruneau and Saxe, 2010; Cikara and Van Bavel, 2014; Hein *et al.*, 2016; Levy *et al.*, 2016; Klimecki, 2019). As such, by implementing the IAT in interdisciplinary and ecologically valid experiments, these two limitations can be mitigated and may open new insights into the connections between implicit intergroup mechanisms and real-life intergroup behaviour.

Although the brain basis of intergroup bias has been investigated for over a decade, studies have often utilized functional magnetic resonance imaging (fMRI) or phase-locked responses in electroencephalography (EEG) (Amodio and Cikara, 2020). By contrast, very little research employed magnetoencephalography (MEG) to investigate intergroup bias [see, however, Levy *et al.* (2016)], and to our knowledge none has employed MEG during IAT. MEG was developed over half a century ago, and it allows to simulate brain activity by recording magnetic fields generated by electrical activity of neuronal populations (Gross, 2019a). There are several physiological constraints, which MEG overcomes, thanks to its capacity to record brain activity directly and non-invasively with a very high temporal resolution. For instance, unlike hemodynamic-based methods (e.g. fMRI and fNIRS), MEG bypasses issues related to intermediate processes (such as neurovascular coupling) (Gross, 2019a), and unlike EEG signal, MEG signal is not distorted by the complex layering of head tissues (Baillet, 2017). Furthermore, whereas fMRI can pinpoint mental processes by relying on the slow cerebral blood flow and phase-locked evoked EEG responses index brief externally driven processes, integrative functions are mainly reflected by sustained rhythmic non-phase-locked activity (Tallon-Baudry and Bertrand, 1999). This type of neural activity is of high relevance in the study of complex social and cognitive processes such as intergroup bias. Non-phase-locked induced activity rhythmically evolves over hundreds of milliseconds and results from intrinsic network interactions within the brain that underpin global processes, such as perception or cognition (Donner and Siegel, 2011). MEG can accurately and non-invasively capture this kind of oscillatory activity and its neural generators (Baillet, 2017; Gross, 2019a), and examining such an activity during the IAT can provide a novel outlook of the neural mechanisms that may underlie the mental processes that are at work during implicit intergroup bias (Levy *et al.*, 2020).

Emotions play a key role in intergroup bias and discrimination (Talaska *et al.*, 2008), and there are various neural rhythms that are implicated in the processing and perception of others’ emotions. For instance, delta and theta rhythms have been implicated in the perception and processing of emotional facial expressions (Balconi and Lucchiari, 2006; Balconi and Pozzoli, 2009; Knyazev *et al.*, 2009a,b, 2010), and emotions that are gated through motivational processes may be associated with theta activity (González-Roldan *et al.*, 2011).The alpha rhythm has similarly been found to underlie the perception of others’ emotions (Balconi and Ferrari, 2012; Gutsell and Inzlicht, 2012; Popov *et al.*, 2013; Meng *et al.*, 2016; Ferrari *et al.*, 2020; Schubring and Schupp, 2020). In general, the mechanism by which the alpha rhythm gates emotional processing is through power suppression (Jensen and Mazaheri, 2010). Furthermore, to our knowledge, alpha rhythm is the only rhythm that has been repeatedly implicated in emotional processes related to intergroup contexts. For instance, alpha suppression has been shown to decrease during the perception of outgroup members experiencing emotions and, to a lesser extent, when subjects were prejudiced (Gutsell and Inzlicht, 2010). The finding that intergroup contexts modulate the level of alpha suppression was replicated in three additional studies on intergroup empathy (Perry *et al.*, 2010; Gutsell and Inzlicht, 2012; Gutsell *et al.*, 2020). More recently, we found that alpha suppression also sustains empathy bias towards conflict group members (Levy *et al.*, 2016), and obvious/automatic associations were found to be underpinned by the alpha rhythm (Di Bernardi Luft *et al.*, 2018).

Evidence consistently suggests that intergroup bias relies on the occipital cortex (Ibáñez *et al.*, 2010; Schiller *et al.*, 2016) or on occipito-frontal connectivity (Forbes *et al.*, 2012). Occipital involvement reflects early automatic/perceptual processes during intergroup bias (Amodio and Cikara, 2020), particularly implicit bias (Marini *et al.*, 2018), and links with the physical characteristics of the stimuli, such as name or face (Marini *et al.*, 2018). Similarly, biased perception is thought to be mediated by occipital attention to outgroup members (Van Nunspeet *et al.*, 2014; Giménez-Fernández *et al.*, 2020). Still, occipital perception is not the only process underlying the bias; phase-locked EEG studies found that late controlled/evaluative processes play an important role in implicit bias (Hurtado *et al.*, 2009; Williams and Themanson, 2011; Hilgard *et al.*, 2013), and that these rely mainly on the middle cingulate cortex (MCC) (Shackman *et al.*, 2011; Healy *et al.*, 2015; Schiller *et al.*, 2016). Schiller and colleagues implemented microstate analysis to define seven processes during implicit bias. Of these, only two processes were found to mirror the bias: one perceptual emanating from the occipital cortex, the right lingual (RL) gyrus, and the second implementing cognitive control and emanating from the MCC. Cognitive control is associated with the ability to regulate emotions (Hendricks and Buchanan, 2016), an ability that is highly critical in intergroup contexts and determines the perpetuation *vs* reduction of intergroup conflicts (Halperin *et al.*, 2011b). Furthermore, given that MCC is a functional hub (Shackman *et al.*, 2011), it is plausible that it may exchange perceptual information with the occipital cortex to guide the controlled biased decision in the IAT. While intergroup bias was found to involve connectivity between occipital and frontal sensors, the specific cortical regions implicated in such connectivity have not been pinpointed (Forbes *et al.*, 2012). Furthermore, the occipital alpha rhythm is involved in perception and attention (Lopes da Silva, 2013); in particular, the literature consistently reports that posterior alpha modulates attention through mechanisms of inhibition and/or facilitation (Peylo *et al.*, 2021). More specific to the current context, the ability of the alpha rhythm to inhibit task-irrelevant neural patterns (Jensen and Mazaheri, 2010) implicates it in selective attention to emotionally salient social stimuli (Symons *et al.*, 2016). Similarly, alpha suppression exerts control over emotional stimuli, which suggests that alpha is involved in control, in addition to perception (Murphy *et al.*, 2020). Hence, the alpha rhythm may be a good candidate to function as a key rhythm in intergroup contexts by controlling the facilitation *vs* inhibition of perceptual and attentional pathways, and previous reports indeed highlight the association between alpha rhythm and intergroup bias (Gutsell and Inzlicht, 2010, 2012; Perry *et al.*, 2010; Levy *et al.*, 2016; Gutsell *et al.*, 2020).

Framed within the Israeli–Palestinian conflict, the current study aims to pinpoint the neural representation underpinning intergroup bias and whether this process is impacted by ingrained beliefs and behaviours that characterize conflicts and impede their resolution. The Israeli–Palestinian conflict has been tearing the region for over a century, leading to violence and suffering among the two major ethnic groups in the region: Jewish and Arabs (Bar-Tal, 2013). The intractability of this conflict impacts perception, behaviour, and attitudes towards the other group, escalating the conflict and impeding positive intergroup dialogue and peaceful settlement (Bar-Tal and Halperin, 2011b). Here, we utilized a cohort of Jewish-Israeli (hereafter labelled Israelis) and Arab-Palestinian (hereafter labelled Palestinians) adolescents. Although the Arab-Palestinians youth in our study are not citizens of the West-bank or the Gaza strip, they are a minority in the state of Israel (ca 21% of the entire population) (Jerusalem Israel, 2021). They typically live in separate neighbourhoods or towns, have minimal opportunities for interpersonal encounters with the majority Jewish group, and share a Palestinian collective national identity (Sammy, 1992).

**Fig. 1.**
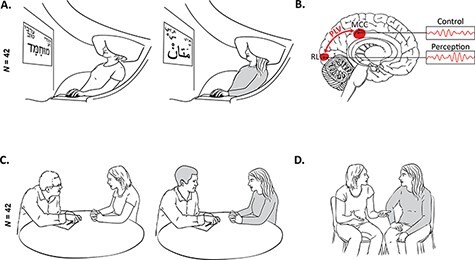
Experimental design and main findings. Upper panels: (A) Jewish-Israeli participants (left) and Arab-Palestinian participants (right) (*N* = 42) alternated to perform the intergroup IAT task in their mother tongue (Hebrew and Arabic, respectively) while MEG continuously monitored neural oscillatory activity. (B) Throughout the whole task, rhythmic reactivity and connectivity in the alpha-band between two key nodes reflect perception and control during intergroup bias. Lower panels: (C) Participants were then interviewed in their mother tongue to measure ‘active belief’, that is, the endorsement of being active for conflict resolution; and conducted (D) an intergroup conflictual to assess ‘active dialogue’, that is, active engagement during the dialogue.

Our study employed a multi-method design and integrated (a) MEG imaging to probe rhythmical activity across a broad spectrum of 1–150 Hz, from the low delta band to the high gamma range, which underpins implicit intergroup bias during the IAT task; (b) lab-based in-depth interviews, and (c) behavioural observation and blind coding of a one-on-one social dialogue between Israeli and Palestinian youth (Figure 1). Our previous work emphasized the importance of dialogue between an Israeli and Palestinian youth and how this can yield positive intergroup relations (Influs *et al.*, 2018, 2019). The interview and social behaviour complemented the neural data by assessing the prominence of an active *vs* passive state of mind and behaviour in the context of the conflict. This shift to being active is crucial as the ever-lasting climate of intractability overshadowing the Israeli–Palestinian conflict tends to result in attitudes of despair and apathy, thereby paralyzing the public and inducing the belief that any action is futile and cannot lead to peace (Halperin, 2016). Moving from a passive state of paralysis to an active state of hope and dialogue is critical for peacemaking (Halperin *et al.*, 2011a; Cohen-Chen *et al.*, 2014). This is particularly relevant during intergroup conflicts as conflicts minimize opportunities for personal encounters between the two groups; hence being actively engaged in encounters for dialogue is paramount for mitigating the devastating outcomes of ongoing conflicts (Ron *et al.*, 2017). Our previous work found that active dialogue can yield positive intergroup emotions and attitudes at the behavioural and hormonal levels (Influs *et al.*, 2018, 2019), and other studies indicated that an active mode of coping with the hardships of conflicts is crucial for the resilience of Palestinian adolescents (Punamäki *et al.*, 2001). Decreased intergroup bias has similarly been associated with active peacemaking (Acar and Uluğ, 2016) and engagement in dialogue (Brannon and Walton, 2013).

Two hypotheses were formulated. First, we exploited the excellent capacity of MEG for distilling cortical rhythmic activity and conjectured that implicit intergroup bias will rely mainly on the alpha rhythm at the integration of the perceptual level, which implicates occipital activity, and the cognitive-control level, implicating cingulate activity; yet, we explored potential effects in the broad 1–150 Hz spectrum. Second, we tested whether our neural proxy for intergroup bias can predict behaviours and attitudes indicating increased levels of active engagement in peaceful dialogue. Specifically, we explored two pathways towards active engagement in real-life dialogue: (i) via perception (occipital alpha): whether this pathway is direct or indirect via beliefs in being active; and (ii) via cognitive control (cingular alpha): whether this pathway is direct or indirect via occipital–cingular information trafficking. Overall, our multi-method study integrated controlled experimentation and ecological paradigms in search of a neural representation underlying intergroup bias and its potential repercussions to real-life behaviour in the context of intergroup conflict.

## Materials and methods

### Experimental design

#### Subjects

Forty-seven healthy Jewish-Israeli and Arab-Palestinian adolescents were recruited for the study via social media, advertisement in schools and in adolescents’ organizations. Participants were right-handed with no serious medical, neurological or psychiatric conditions and were all MEG-compatible (mainly metal-free, for instance, free of tooth bracelets, implants, piercing, etc.). Following data acquisition, five participants were excluded: three due to averaged head-position deviations exceeding coregistration-error threshold (1 cm) and two due to failure of completing the MEG paradigm. This resulted in a cohort of 42 participants, 57.14% Arab-Palestinians and 52.38% males, ranging in age from 15 to 18 years (*M* ± s.d., 16.49 ± 0.83). The study received approval from the Bar-Ilan University ethics committee, and participants’ parents gave written informed consent before the experiment in line with the Bar-Ilan University’s Institutional Review Board, and youths were informed that they can leave at any point during each session or drop out of the study and received monetary compensation for their participation. Adolescents were residents in the centre of Israel (within a 50 km distance from Tel-Aviv) with religiousness levels similar to the proportions within the general population (CBS Jerusalem Israel, 2021).

#### Behavioural measures

*Active Dialogue*. To evaluate adolescents’ active engagement towards an outgroup member, we utilized a validated ‘conflict dialogue’ paradigm (Schneiderman *et al.*, 2014); this is a one-on-one discussion between same-sex mixed-group partners, one Jewish and one Arab-Palestinian, randomly assigned (Levy *et al.*, 2016). We asked the dyads to discuss about a conflictual topic (e.g. national or personal) of their choice for about 10 min. The dyadic interaction then underwent coding offline by external coders who were unaware of the experimental conditions and the purpose of the study. Consistent with previous research (Feldman, 2021), coding relied on the well-validated Coding Interactive Behaviour (CIB) manual, and we then averaged five CIB items to convey the *active dialogue* construct: enhanced involvement, motivation and persistence, reduced hostility, mirroring or withdrawal. Rating was completed according to a 5-point scale ranging from 1 (none) through 2 (little), 3 (moderate), 4 (high) to 5 (very high) for each one of the five items.

#### Active belief

We conducted an in-depth structured interview with each participant. A qualitative–quantitative transformation was then conducted by the interviewers by rating participants’ attitudes towards each item on a 3-point scale ranging from 1 (strong opposition) through 2 (weak endorsement) to 3 (full endorsement). We then computed a measure of ‘*active*  *belief*’ (Table 1) by averaging five items that describe the degree to which participants endorsed being active as a means for conflict mitigation/resolution.

**Table 1.** The ‘active belief’ construct

Jewish-Israelis	Arab-Palestinians	Both sides
Education, territorial concessions and learning more about the conflict	Education, making compromises and leading towards peace	Avoiding passivity
Stopping violence and racism	Stopping violence	

The five items that were used to compute the ‘active belief’ score delineate the specific active steps that need to be made (by each one of the two camps, or by both) in order to achieve conflict mitigation.

#### IAT neuroimaging stimuli

Participants completed the IAT (Greenwald and Lai, 2020) in the MEG scan, and IAT stimuli were either in Hebrew or in Arabic, as a function of participants’ mother tongue. Stimuli were generated using the E-prime software (Psychology Software Tools Inc.) in a dimly lit room and presented in the centre of the screen in Courier New, size 12, so that stimuli are foveally presented (horizontal visual angle < 2.5^°^). A black background projected through a mirror on an LCD monitor placed at a viewing distance of 50 cm. Responses were delivered by a response pad. A photosensitive diode on the screen recorded the onset time of visual stimuli. Stimuli were 40 frequent given names (Arab/Jewish/male/female; length: 3–7 letters) and 40 frequent words with a positive or negative valence (length: 3–7 letters; usage frequency 2–50 per million (Frost and Plaut, 2005)); the stimuli were validated before the study for group membership by 12 independent Arab-Palestinian and Jewish-Israeli raters. These stimuli were used exclusively for the two test blocks while 40 additional stimuli were generated for the practice blocks. Stimuli were presented on a black background to avoid ocular fatigue while reminder labels of stimulus categories (Arab/Jewish, good/bad) were present on the bottom of the screen only during the practice blocks so as to avoid eye movements during the test blocks. Names were in grey lowercase letters, while words were presented in yellow lowercase letters.

#### IAT design

A general IAT design was applied (Greenwald and Lai, 2020) with minor modifications and addition of blocks so as to optimize the design for MEG recording as detailed below. IAT measures implicit negative associations towards preselected targets (e.g. outgroup members) and it relies on contrasting trials with C *vs* IC implicit associations. Assuming negative implicit association towards outgroup members, for Jewish/Arab participants, the mapping of name and word discriminations to the response buttons was stereotype congruent (e.g. left button press to an unpleasant word or Arab/Jewish name, and right button press to a pleasant or Jewish/Arab name), respectively. In IC trials, this mapping is reversed, that is, left button press to a pleasant word or Arab/Jewish name, and right button press to an unpleasant or Jewish/Arab name. Behavioural responses were generated by pressing with either the index or the middle fingers on a response pad, corresponding to each side of the screen, thereby targeting either of the IAT categories. There were 10 experimental blocks, 8 practice and 2 test blocks, and each began with a brief explanation of which classification category, or category combination, was assigned to each response key. In a nutshell, in block 1, participants categorized names either as Arab or as Jewish (20 trials); in block 2, they were asked to categorize words as either good or bad (20 trials); in block 3, intergroup and evaluative categories of blocks 1 and 2 were pooled together in a single task of combined classification, such that stimuli were categorized, for example, as Arab—positive and Jewish—negative (20 trials). This discrimination task was carried out in practice block 3 and repeated in block 4 with covert category labels to reduce eye movements as preparation for the test block; block 5 was the C test block and consisted of 160 trials. Then, blocks 6–10 were repeated with different stimulus order and this time with a reversed congruency manipulation, so that block 10 was the IC test block. Hence, in comparison to typical IAT designs, we added additional practice blocks (i.e. blocks 4 and 9) with covert category labels to reduce eye movements as the preparation for the test block and then analysed data from the two test blocks that do not require eye movements for the completion of the task, thereby distilling as much as possible bias-specific activity. The order of C/IC blocks and the sequence of names and words were counterbalanced across subjects. Trials from the two test blocks yielded two matched experimental conditions, IC and C blocks, comprising a total of 320 trials while measuring participants’ brain activity, resulting in duration for approximately 20 min, including breaks.

#### Neuroimaging experimental procedures

Participants lay in supine position inside the MEG system while facing a screen projecting the stimuli. Subjects received instructions to remain relaxed and not move their limbs; the experimenter observed their compliance using an infrared camera. IAT stimuli were presented until response key was made, interleaved with crosshair fixation screens randomly varying in duration between 1170 and 1670 ms. Participants were notified when an error was made (i.e. assigning word/name in the opposite category), and they were asked to correct their response; these error trials were not included in the neural data analysis so as to avoid interfering processes (e.g. motor movement, surprise, self-correction).

#### MEG recordings

We recorded ongoing brain activity (sampling rate, 1017 Hz, online 1–400 Hz band-pass filter) using a whole-head 248-channel magnetometer array (4-D Neuroimaging, Magnes® 3600 WH) inside a magnetically shielded room. Reference coils located approximately 30 cm above the head, oriented by the *x*, *y* and *z* axes, enabled the removal of environmental noise. Movements were visually monitored by the experimenter via a camera and by a movement-tracking system using five coils attached to the participants’ scalp to record the head position relative to the sensor array. Under-aged populations move inside the MEG quite often (Phillips, 2005); hence, we determined the threshold for movement to 1 cm as we previously applied (Levy *et al.*, 2019). Three out of the 45 adolescents yielded averaged deviations exceeding that threshold, and were therefore not included in source analysis so as to minimize coregistration errors and to maximize source precision.

#### Data preprocessing and MEG sensor-level analysis

Four steps aimed to clean artefacts and noise: (i) we removed external noise (e.g. power-line, mechanical vibrations) and heartbeat artefacts from the data using a predesigned algorithm for that purpose; (ii) we rejected trials containing muscle artefacts using visual inspection; (iii) we removed eye-blinks, eye movements, or any other potential noisy artefacts using spatial component analysis (ICA); and (iv) a final visual inspection of every trial verified any other noise/artefact to be removed from further analysis. We excluded two sensors from the analysis due to malfunction. We segmented the data into 2500 ms (baseline period:500 ms) epochs corresponding to the IAT event trials in alignment to stimulus onset and retained for analysis only trials with a response time longer than 300 and shorter than 3000 ms, following IAT analysis recommendations (Greenwald *et al.*, 2003). This approach enables examining perceptual and cognitive control processes, which typically occur in the first 1000 ms, while maximizing statistical power by retaining trials with response times that are superior to 2000 ms and inferior to 3000 ms. Epochs were filtered at 1–200 Hz range with 10 s padding and resampled to 400 Hz.

We performed analyses using MATLAB 7 (MathWorks®, Natick, MA, USA) and the FieldTrip software toolbox (Oostenveld *et al.*, 2011). We first computed the typical *D*ʹ index, a behavioural index of the IAT effect, taking into account the difference in response time between the IC and C conditions and the number of errors made, according to IAT analysis guidelines (Greenwald *et al.*, 2003). Data were then analysed in alignment to stimulus onset and time–frequency representations (TFRs) of power were computed while calculating the fast Fourier transform (FFT) for short sliding time windows, and power estimates were averaged across tapers. For the 1–40 Hz frequency range, a Hanning taper was applied to each epoch of the sensor data, yielding the FFT for a short sliding time windows of 0.5 s and a spectral resolution of 2 Hz. For the 40–150 Hz range, five Slepian multitapers were applied using a fixed window length of 0.2 s, resulting in a frequency smoothing of 15 Hz. We obtained induced activity by subtracting evoked components’ power from oscillatory power.

#### Head grid, source reconstruction and connectivity

We divided the subject’s brain volume into a regular grid, obtaining the grid positions by their linear transformation in a canonical 1 cm grid. This procedure facilitates group analysis, because it requires no spatial interpolation of the volumes on reconstructed activity. For each grid position, we reconstructed spatial filters with the aim of optimally passing activity from the location of interest, while suppressing activity that was not of interest. We computed the cross-spectral density matrix between all MEG sensor pairs from the Fourier transforms of the tapered data epochs. We constructed spatial filters for each grid location, based on the identified frequency bin, and projected the Fourier transforms of the tapered data epochs through the spatial filters. We built a single shell brain model based on an Montreal Neurological Institute (MNI) post-puberty template brain (Fonov *et al.*, 2011), which we modified to fit each subject’s digitized head shape using SPM8 (Wellcome Department of Imaging Neuroscience, University College London,www.fil.ion.ucl.ac.uk). Head shape underwent manual digitization (Polhemus FASTRAK® digitizer). We applied adaptive spatial filtering (Gross *et al.*, 2001) relying on partial canonical correlations and the cross-spectral-density matrix was computed between all MEG sensor pairs from the Fourier transforms of the tapered data epochs at the statistically significant time–frequency sensor pattern. Finally, we extracted time series from the localized source in the alpha-band by applying a linear constrained minimum variance beamformer that were used for two purposes: calculating and plotting time–frequency analysis, and conducting phase locking value (PLV) connectivity analysis. The latter is a measure of phase synchrony across voxels, thereby reflecting functional communication between brain regions (Lachaux *et al.*, 1999). This was based on a seed-region approach: PLV was computed between the seed that was previously localized via the partial canonical correlations beamformer, and each one of the other grid locations throughout the head grid. Non-parametric statistical testing (cf, below) determined *t*-values per each one of the seed-grid connections, and then a cluster-based correction for multiple comparisons was applied.

### Statistical analysis

To test for mediation, we used contemporary practices of the simple linear mediation model by Hayes (2013) using PROCESS macro. Unstandardized indirect effects were computed for each 10 000 bootstrapped samples, and the 95% confidence interval was computed by determining the indirect effects at the 2.5th and 97.5th percentiles. We estimated the conditional effect of the independent variable ‘intergroup perception’ (X) on the outcome variable ‘active—dialogue’ (Y), with active—belief as a mediator (M). The PROCESS macro for SPSS (v. 2.1.3.2) Model 6 was utilized for this analysis. PROCESS employs bootstrapping calculations, a nonparametric resampling procedure, which provides the most powerful and reasonable method of obtaining confidence limits for specific indirect effects. For these analyses, bias-corrected standard errors and confidence intervals were generated using 10 000 bootstrapped samples. Mediation is considered present when the confidence interval for the estimation of the indirect effect does not contain zero. Statistical power and effect size calculations were achieved using G*Power software (Faul *et al.*, 2007). In all statistical comparisons, we applied an independent two-sided *t*-test. Further, statistical procedures on the MEG data assessed the significance of the power values using a randomization procedure to obtain correction for multiple comparisons (Maris and Oostenveld, 2007). The full statistical details of that cluster-based nonparametric permutation approach is elaborated in our previous publications (Levy *et al.*, 2018, 2019).

## Results

Figure 1 illustrates the experimental design and the main MEG findings. Forty-two adolescents, either Arab-Palestinians or Jewish-Israelis, performed the IAT in their mother tongue while we measured ongoing oscillatory neural activity using MEG. They also performed a task of in-depth interview each one in their mother tongue and then a dyadic intergroup dialogue.

### Intergroup bias at the behavioural level

As expected, during the IC and C conditions, participants responded significantly (*P* = 8 × 10^−8^, *t*(41) = 6.51) slower in the first (*M* ± s.d., 1005.30 ± 178.97 ms) compared to the second (*M* ± s.d., 865.93 ± 145.32 ms) condition, indicating an IAT effect at the behavioural level. The second expected pattern of behavioural results was that the IAT effect was reversed as a function of the ethnic group, that is, Jewish-Israelis had negative implicit associations towards Arab-Palestinians and vice versa. Hence, absolute values were applied for the calculation of *D*, which was thereby significantly (*P* = 2 × 10^−13^, *t* = 8.71) larger than zero (*M* ± s.d., 0.40 ± 0.31), further consolidating the behavioural IAT effect. There was no statistically significant difference (*t*(40)* = *−1.16, *P = *0.25) between ethnic groups on this behavioural effect.

### Intergroup bias is expressed at the level of perception in the alpha rhythm

To look at neural rhythmic activity underlying intergroup bias, we followed by computing the TFRs of each of the conditions and of the contrast between the IC and the C condition, which similarly to the *D*ʹ is thought to reflect the IAT effect at the ‘neural level*’*. Rhythmic activity was examined in the broad 1–150 Hz spectrum. As the functional mapping of alpha-band activity is broad and encompasses motor and various other cognitive and affective processes (Levy *et al.*, 2013, 2018, 2019), we proceeded with distilling alpha functionality to the intergroup bias in a robust analysis pipeline (Levy *et al.*, 2013) as follows. First, the MEG sensor array detected that the neural response during IC and C trials, compared to the baseline period, was expressed as alpha (7–11 Hz) and beta (14–28 Hz) suppression; there was no significant effect (*P* > 0.13) at the higher frequencies spectrum (30–150 Hz). More importantly, to tap into the IAT neural effect, we contrasted the IC and the C conditions, yielding very robust sustained alpha-band suppression throughout the epoch trials (and particularly from stimulus onset onwards). There was also a later beta-band suppression beginning on average around 700 ms post stimulus onset, and peaking above left centro-anterior sensors at ∼ 900–1400 ms (*P*_cluster-cor_ < 0.01). Source reconstruction revealed that this activity emanates from the left sensorimotor cortex (*P*_cluster-cor_ < 0.001) thereby most probably reflecting the contralateral motor activity involved during the right-hand button response, which occurred around that time (*M* ± s.d., 935.62 ± 176.55 ms), as typically observed in previous studies (Donner *et al.*, 2009). Second, in order to distill activity that is as specific as possible to intergroup bias, we excluded motor and response preparation activity (earliest individual response time was 565 ms) as well as pre-perceptual activity, thereby focusing on the time-window at ∼100–550 ms; analysis revealed that alpha activity at that time-interval peaked above right centro-posterior sensors.

**Fig. 2.**
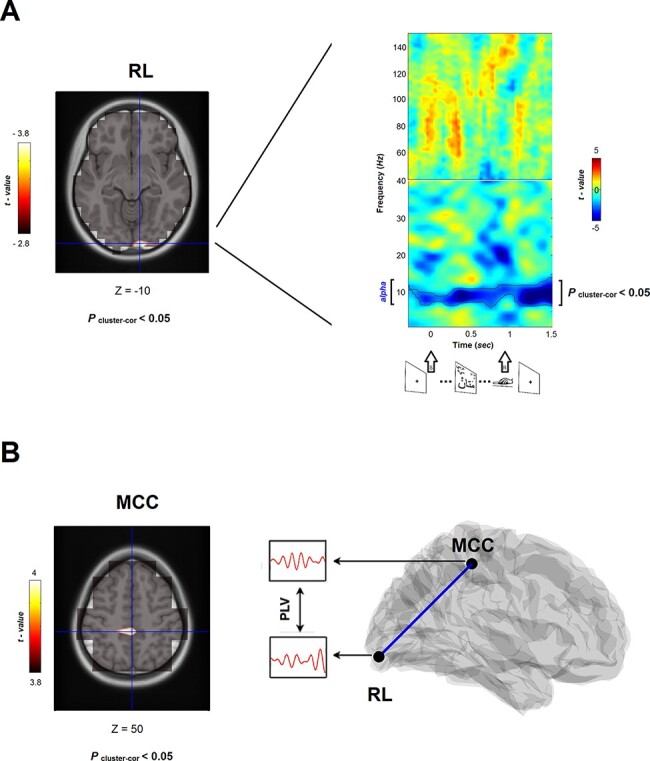
Neural representations of intergroup bias. (A) On the left, an overlaid cortical axial (MNI template) representation of the intergroup bias perceptual contrast illustrates a significant cluster (*P*_cluster-cor_ < 0.05; colour bar illustrates masked significant clusters) in the RL gyrus; on the right, TFR maps of induced oscillatory activity (1–150 Hz; −0.4–1.5 s) filtered from the RL peak coordinates (i.e. virtual channel) illustrate that across the 1–150 Hz, significant rhythmic activity (*P*_cluster-cor_ < 0.05 in contoured patterns) emerges only in the alpha range. Time axis and insets below illustrate that stimuli appeared at zero and were replaced by fixation cross once response decision was made (*M* ± s.d., 935.62 ± 176.55 ms). (B) On the left, an overlaid cortical axial (MNI template) representation of phase-lag-value (i.e. PLV) connectivity from RL as seed region (*P*_cluster-cor_ < 0.05; colour bar illustrates masked significant clusters) revealed the MCC, thereby characterizing information trafficking between RL and MCC as reflecting another representation of intergroup bias (right).

Hence, these analyses revealed that activity in the alpha band (extending to the high theta band) is the main frequency-band candidate associated with intergroup bias, without a statistically significant difference (*t*(40) = 1.14, *P* = 0.26) between the two ethnic groups. We then proceeded to localizing the neural substrates characterizing the IAT effect (IC *vs* C); the alpha effect was localized in the RL gyrus, a hub in the occipital/visual cortex (Figure 2) with a medium-large effect size (Cohen’s *d* = 0.59) and robust statistical effect (*P*_cluster-cor_ < 0.05; 98% statistical power). Time series were then extracted from this cortical patch and a permutation test on each time sample was conducted while contrasting the two conditions (IC *vs* C) (Figure 2, right panel). There was no statistically significant difference (*t*(40) = 1.35, *P* = 0.18) between ethnic groups on this source effect. The alpha effect in the RL was sustained throughout the trial (*P*_cluster-cor_ < 0.05) (Figure 2), and we were interested to test whether there was a time window during which the effect was strongest. The peak of the effect was between 300 and 500 ms, and this response was significantly (*P* < 0.05) more robust than during the baseline period, underscoring a sustained effect that peaked at the perceptual (i.e. visual perception of the word stimuli) time window. Further, we were intrigued whether the effects may have been affected by habituation to the task, that is, whether the neural effect was similar at the beginning compared to the end of the task. We therefore pooled apart the trials into two, and tested the neural effect in both pools. The analysis revealed that both pools reflected the alpha effect with no statistically significant (*P* = 0.43) effect between the two. This rules out the possibility that perceptual habituation may have affected the neural intergroup effect.

### Intergroup bias is expressed at the level of cognitive-control in the alpha rhythm

Further, to explore information trafficking, we conducted a seed-connectivity analysis from the RL source and investigated the other sources to which it is communicating at the alpha band while reflecting the IAT bias (stronger connectivity in the IC *vs* C trials). The results revealed two significant peak nodes (*P*_cluster-cor_ < 0.05) in the cerebral cortex (and an additional source in the cerebellum): the left-lingual gyrus and the middle cingulate cortex (MCC) (Figure 2). Because the first peaks likely reflects the extent of perceptual processes during the IAT task (inter-hemispheric visual processing (Stephan *et al.*, 2007), we were more interested in exploring the connectivity between RL and the MCC as we initially introduced that the two regions reflect perceptual and controlled processed during the IAT, respectively (Schiller *et al.*, 2016)). The RL–MCC connectivity reflecting the intergroup bias yielded a statistically robust (*P* = 0.00007; 99% statistical power) difference between PLV values in the C (*M* ± s.d., 0.3551 ± 0.2875) and the IC (*M* ± s.d., 0.4043 ± 0.3044) conditions, with a relatively large effect size (Cohen’s *d* = 0.68). Time series were then extracted from this cortical patch and a permutation test on each time sample was conducted while contrasting the two conditions (IC *vs* C). These revealed that cortical reactivity at this neural patch was expressed as alpha suppression, particularly between 1.1 and 1.5s, thereby further supporting the assumption that the MCC reflects late cognitive control, as opposed to the early perceptual processing in the RL. There was no significant difference between the two ethnic groups on the connectivity measure (*t*(40)  *<* −1.62, *P > *0.12). These findings confirm our first hypothesis that intergroup bias relies on the alpha rhythm and integrates the perceptual (implicating occipital activity) and cognitive-control (implicating cingular activity) levels.

### Neural representations of intergroup bias predict intergroup dialogue style

**Fig. 3.**
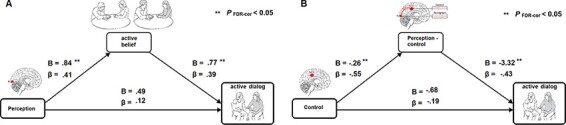
Mediation analyses. Upper triangle: the first mediation analysis revealed that perception (RL activity) leads to ‘active belief’ (*P*_FDR-cor_ = 0.0077; model summary = 0.0077), which leads to active dialogue (*P*_FDR-cor_ = 0.02; model summary = 0.01), while perception does not significantly lead to active dialogue (*P*_FDR-cor_ = 0.46; model summary = 0.01). Lower triangle: the second mediation analysis revealed that control (MCC activity) leads to perception-control (RL–MCC connectivity) (*P*_FDR-cor_ = 0.0002; model summary = 0.0002), which leads to active dialogue (*P*_FDR-cor_ = 0.02; model summary = 0.06), while control does not significantly lead to active dialogue (*P*_FDR-cor_ = 0.30; model summary = 0.06).

Having identified these neural signatures of intergroup bias, that is, alpha rhythmic modulations at the RL, MCC and the connectivity between them, we next examined how these neural effects predict increased levels of active engagement in intergroup dialogue (i.e. ‘active dialogue’). A first pathway towards this behavioural outcome was explored: whether perceptual bias leads to active dialogue directly or indirectly via believing in being active as venue towards peace (i.e. ‘active belief*’*). To this end, we observed adolescents’ active engagement during one-on-one dialogue with an outgroup member, and next, using an in-depth interview, we measured the degree to which adolescents perceived the belief in being active as venue for peace (see the ‘Methods’ section). The two groups revealed a rather high level of ‘active dialogue’ (on a scale of 1–5: (*M* ± s.d., 4.16 ± 0.55)) during outgroup dialogue and expressed a medium level (on a scale of 1–3: *M* ± s.d., 1.94 ± 0.28) of ‘active belief’, with no significant difference between the two ethnic groups on these two measures (*t*(38) < 0.70, *P* > 0.49). As outlined in our hypothesis section, a first mediation analysis revealed that ‘active belief’ mediated (*P* < 0.05) the effects of intergroup perception as captured by occipital alpha (RL) on ‘active dialogue’, and that the two latter variables were not directly linked (*P* = 0.46) (Figure 3). Next, a second pathway towards active engagement in intergroup dialogue was explored: whether the cognitive control neural component (MCC) in the bias leads to ‘active dialogue’ directly or indirectly via perception-control information trafficking (reflected by RL–MCC neural communication). The second mediation analysis thus revealed that perception-control (RL–MCC) mediated (*P*_FDR-cor_ < 0.05) the effects of biased control (MCC) on ‘active dialogue’, and that the two latter variables were not directly linked (*P* = 0.30) (Figure 3). Altogether, these analyses confirmed the second hypothesis by charting two separate neural pathways of intergroup bias, one perceptual and the next controlled, both of which indirectly lead to active engagement in intergroup dialogue.

## Discussion

Intergroup bias is a puzzling phenomenon: humans’ evolutionary-based reliance on group living has led to systematic inaccuracies in the perception and judgment of one’s own social group in comparison with other groups (Hewstone *et al.*, 2002). While such skewed perceptions have paved the way for the consolidation of group living across human evolution, they also carried devastating consequences, from discrimination to violent intractable conflicts (Bar-Tal and Halperin, 2011a). Despite its key impact on the processes of social perception and cognition, capturing the neural underpinning of intergroup bias remains challenging (Amodio and Cikara, 2020). Our study uniquely utilizes MEG to tap the neural underpinnings of intergroup bias and to exploit its special ability to examine multiple intrinsic neuronal rhythms and their interactions across the cerebral cortex. Findings provide convincing evidence that highlights alpha as a mechanism that integrates intergroup bias into one intrinsic and sustained process, from the early stages of perception, via the occipital cortex, to the later top-down cognitive control, via the cingulate cortex. Furthermore, our study answers the recent call made by intergroup neuroscientists to connect basic neurocognitive processes with real-world phenomena (Amodio and Cikara, 2020). While recent studies provide important insights into the neural substrates of intergroup conflicts by simulating conflict during computer games (Han *et al.*, 2020; Yang *et al.*, 2020), we capitalized on the ‘natural experiment’ of the Israel–Palestinian conflict and its long history of intense bias and inflexible attitudes (Halperin, 2016). By integrating MEG imaging with naturalistic paradigms of intergroup dialogue and in-depth interviews of Israeli and Palestinian youths, we investigate how neural mechanisms can explain real-world attitudes and behaviour associated with the conflict. We show that intergroup bias is reflected by the alpha rhythm and integrates perceptual bottom-up processes with controlled top-down mechanisms to predict real-world behaviour and attitudes of youth in the two sides of the conflict.

The brain is organized in a system of rhythms, with repeating patterns in time and space providing a mirror for mental reality (Buzsaki, 2004; Fries, 2015). Yet, for a long period, the localizationist approach to mental functions dominated scientists’ vision of brain function. Emerging in the late nineteenth century, neurologists and anatomists such as Paul Broca consolidated the attribution of function to specific brain areas. A century later, hemodynamic neuroimaging techniques (e.g. fMRI) renewed the localizationist approach by identifying one-to-one mapping between cognitive functions and statistical brain maps; yet, with time it became apparent that this approach is too simplistic. By contrast, the rhythmic view of the brain function shifts the focus from substrate to frequency. Synchronous oscillations correspond to the rhythmic patterns of neural activity at multiple frequencies, facilitating intrinsic functional networking (König and Schillen, 1991; Hutt *et al.*, 2016) and enabling a given neuronal pool to contribute to different cognitive functions at various time scales. Synchronous rhythms play a critical role in linking areas that are part of the same functional network and can probe representations, which may be overlooked by the slow hemodynamic response, and thereby reflect the interplay between bottom-up (e.g. perception) and top-down (e.g. cognitive control) mechanisms (Tallon-Baudry and Bertrand, 1999; Bastos *et al.*, 2015). MEG can effectively disentangle task-specific from non-specific perceptual processes by yielding frequency-based representations in simultaneous active brain regions (Whitmarsh *et al.*, 2011; Levy *et al.*, 2014; Baillet, 2017; Gross, 2019b). Our study, the first to apply MEG to study the neural underpinnings of intergroup bias, may pinpoint a novel oscillatory lengthy process that integrates its perceptual and cognitive components.

Two brain regions were found to underpin the intergroup bias, the RL and the middle cingulate cortex (MCC). These results are consistent with those of Schiller and colleagues, who conducted microstate EEG analysis and found that the only two regions that plausibly reflected intergroup bias activity are the RL and the MCC/PPC, which occurred early and late, respectively (Schiller *et al.*, 2016). However, their analysis relied on assessing IC (i.e. outgroup associated with good) and C (i.e. ingroup associated with good) responses and pinpointing the EEG components that took longer to process in IC *vs* the C trials. While this approach is informative, it relies on the representation of brief, separate, and externally driven processes and lacks the integration of the observed responses into a prolonged and intrinsic representation. Still, complex mental processes that sustain the intergroup bias likely rely on intrinsic network interactions that repetitively evolve and communicate over hundreds of milliseconds, as conveyed by induced oscillations (Donner and Siegel, 2011). Our study implements this approach by directly capturing the intergroup bias while contrasting IC with C trials and assessing induced activity throughout the entire task. Our findings show that the two regions—RL and MCC—do not operate separately but function within a single rhythm, the alpha rhythm, which is sustained throughout the entire task while trafficking information between the two nodes. The first node, the RL and the occipital cortex in general, is critical for the perceptual component of intergroup bias and involves visual processing of the stimuli’s physical properties, such as names, faces, or visual representations of group members (Marini *et al.*, 2018). The second node, the MCC, constitutes a hub where the perceptual information is linked to motor centres responsible for affect and goal-directed behaviour (Shackman *et al.*, 2011). This can bias responses when the most adaptive course of action is uncertain, for example, while controlling automatic responses to outgroup representations (Van Nunspeet *et al.*, 2014). By demonstrating rhythmic integrative network operations that integrate bottom-up and top-down processes of intergroup bias, our findings go one step beyond the findings of individual microstate, phase-locked, or anatomically constraint events. Our aim was to capture the global phenomenon while critically revealing the neural underpinnings of intergroup bias, and our findings demonstrate that intergroup bias shifts the brain’s alpha rhythmic activity as a prolonged state that involves both perceptual and controlled components.

Our finding that intergroup bias relies on alpha rhythm is in line with several previous EEG studies that measured averaged alpha power in EEG channels during the perception of outgroup *vs* ingroup characters in movement (Gutsell and Inzlicht, 2010), pain (Perry *et al.*, 2010), or sadness (Gutsell and Inzlicht, 2012). Although these studies do not provide examination of induced rhythmic activity across time, multiple rhythms, or cortical generators, they are informative in confirming the involvement of the alpha rhythm in the biased intergroup perception. While we maintain that the neural underpinning of the intergroup bias charts a single sustained neural process, the question remains as to whether intergroup bias is reflected solely by the alpha rhythm, or additionally by other neural rhythms. Notably, while the question of neural specificity cannot be verified here, we examined the entire spectrum of rhythms from 1 to 150 Hz and found that alpha was the main rhythm continuously involved in the process. Noteworthily, another rhythm that was revealed in this study is in the beta band; yet, our analyses found that the beta effect was generated by the sensorimotor cortex and was contralateral and concurrent in timing to the response press. It is therefore plausible that it was involved in sensorimotor processing. Importantly, MEG is advantageous in dissociating the functionality of neural rhythms, as we found in a previous study with a different task, which associated sensorimotor activity to the beta rhythm and perceptual and cognitive processes to the alpha rhythm (Levy *et al.*, 2013). Likewise, extant evidence suggests that beta reflects neural reverberation that mediates persistent activity during sensory evidence -accumulation before motor decision (Donner and Siegel, 2011; Alavash *et al.*, 2017). We are aware of only one study reporting the involvement of beta and not alpha rhythm in intergroup bias while showing videos of painful actions (Riecanský *et al.*, 2015). In that EEG study, sensory and motor involvements were intertwined and the authors expressed the difficulty of dissociating them, which is almost impossible in EEG studies. While that study looked at EEG channel data rather than cortical sources, the capacity of MEG to dissociate the function of targeted cortical rhythms enabled us here to interpret the beta activity in the current study as most plausibly sensorimotor rather than perceptual or cognitive.

Our findings in the theta rhythm show that the alpha response is quite broad and extends to the high theta band. Such a spectral extension is quite often observed, for instance, in empathy to vicarious pain (Levy *et al.*, 2018), to vicarious emotional distress (Feldman *et al.*, 2019), and even to intergroup empathy (Levy *et al.*, 2016). It is noteworthy that the boundaries between frequency bands are not fixed by definition and are arbitrarily determined. As such, while it is quite unlikely that the low-alpha observed here is a distinct theta response, one possible interpretation of the findings is that the low-alpha activity may include concurrent high-theta functioning, particularly in the later cognitive control phase from more anterior cortical sources, given the implication of frontal theta in cognitive control (Cavanagh and Frank, 2014). However, we suggest that the alpha effect reported here reflects alpha functioning rather than theta–alpha due to the pronounced mechanistic difference between the two bands; when the function is to relay information and recruit neural substrates (e.g. for cognitive-control), alpha power is typically suppressed (Jensen and Mazaheri, 2010), whereas theta power is typically enhanced (Cavanagh and Frank, 2014; Cooper *et al.*, 2019; Duprez *et al.*, 2020). Altogether, the current effect mainly centres in the alpha range and is clearly suppressed and most probably reflects alpha functioning.

An interesting association revealed in this study was between the neural representations of intergroup bias and key behavioural (i.e. ‘active dialogue’) and attitudinal (i.e. ‘active belief’) measures. These measures reflect a mental climate of being active *vs* the passive stance that characterizes the Israeli–Palestinian conflict (Halperin, 2016). Our results report that the neural underpinnings of the intergroup bias may lead to a passive dialogue style among youths of the two sides, and, conversely, individuals from the two ethnic groups who had decreased levels of neural intergroup bias conducted intergroup dialogue more actively (i.e. ‘active dialogue’). Although causality cannot be inferred, our two mediation analyses provide an interesting perspective. As seen, the two aspects of the intergroup bias, perception and cognitive control, each resulted in a passive mode of dialogue via two different pathways (Figure 3): The first pathway shows that moderating the perceptual component of the bias is the individual’s belief in the importance of being active for peacemaking and intergroup conflict resolution. Thus, explicit beliefs of active peacebuilding shape the way implicit neural perceptions impact dialogue with outgroup members. The second pathway shows that moderating the effects of the control component on an intergroup dialogue is neural coordination between the first perceptual and control components of the neural underpinnings of the bias. Such neural connectivity mediates the relations between the top-down control and dialogue with outgroup members. These findings highlight how disentangling the neural representation of intergroup bias into its components can shed further light on their specific links with dialogue behaviour in the context of intractable conflict spanning several generations.

Our results connecting neural mechanisms with a real-life behaviour may contribute to the ongoing debate on whether the IAT can predict real-world intergroup behaviour (Oswald *et al.*, 2013). One possibility is that relying on a single neural mechanism, instead of multiple non-specific EEG and fMRI signatures, may yield more consistent associations between IAT and behaviour. Recently, evidence points to the ability of neuroimaging data to quantitatively assess various cognitive and affective processes and to predict outcomes better than traditional behavioural and self-reported measures (Gabrieli *et al.*, 2015). Hence, good neuroimaging paradigms can successfully yield objective, specific, and quantitative measures (Bruneau, 2015; Hein *et al.*, 2016). Previous neuroimaging studies on the IAT have focused on the mechanisms involved in the IAT rather than on its real-world implications (Ibáñez *et al.*, 2010; Williams and Themanson, 2011; Forbes *et al.*, 2012; Hilgard *et al.*, 2013). Here, we found that the neural measures linked with real-world situations, despite the fact that it is very challenging to empirically evaluate intergroup behaviours in real-life settings (Amodio and Cikara, 2020) using naturalistic lab-based paradigms to emulate it successfully (Levy *et al.*, 2020). Our empirically tested and validated approach to emulating real-life intergroup behaviour is via lab-based dialogue between members of two conflicting groups, which is then analysed for its verbal and non-verbal signals (Levy *et al.*, 2016; Influs *et al.*, 2018, 2019). The current findings extend previous work and show that the alpha rhythm is a neural underpinning of intergroup bias and additionally predicts real-world dialogue.

Several points should be taken into consideration in the interpretation of the findings and their limitations. First, despite the rigor and complex methods and design implicated in this study, the paradigms did not include no-task control conditions, nor did the study use any manipulation that may have yielded causal inferences. Future studies on intergroup bias should implement both no-task control conditions and non-invasive brain stimulation to further probe the centrality of the alpha rhythm and its plausible causal role in intergroup bias. Second, previous works have shown that cognitive control linearly improves with age; adolescents’ emotion regulation is weaker than adults’ (Tottenham *et al.*, 2011), cognitive control in adolescence is achieved via the down-regulation of task-irrelevant information (Banich *et al.*, 2019), and the neural intergroup bias can be found in a vicarious reward task during adolescent development (Brandner *et al.*, 2021). This is noteworthy given that the current study sampled adolescents. One can therefore speculate that in a sample of adults or in children, the increase in the ability to control and to regulate emotions might implicate different neural mechanisms. Our previous works showed that alpha rhythm changes throughout development when generating empathy to vicarious suffering (Levy *et al.*, 2018); hence, future cross-sectional developmental studies would further expand the current body of knowledge on the neural underpinnings of intergroup bias. Third, the present study is very challenging to conduct: recruiting adolescents from two rival ethnicities under a climate of conflict to a single location while complementing real-life interactions and in-depth interviews and thus, our sample is not very large. We therefore advocate that future studies reproduce these findings implement larger samples. Still, the reliability of the neural mechanisms reported here, in terms of both alpha reactivity and alpha connectivity, is very high (>97% statistical power) and aligns with the call for credibility in published scientific studies (Munafò *et al.*, 2017). Finally, because oscillations in the brain develop on the basis of prior experiences and maturity (Levy *et al.*, 2018), future directions may explore whether this neural mechanism shifts across development and whether it is sensitive to education, social construal and conflict. Given that the neural representations reported here links with intergroup dialogue and beliefs, they pinpoint targets for youth interventions that may mitigate intergroup bias, while providing opportunities for one-on-one encounters with outgroup members, and foster the value of being active even in times of violence. We hope that the approach presented here can motivate social neuroscientists to extend their investigations to neural rhythms that underlie our social life and its implications with real life attitudes and behaviour that foster active engagement in peaceful dialogue.
